# CXCR2 associated neutrophil stromal interactions and immune exclusion in microsatellite stable colorectal cancer

**DOI:** 10.3389/fimmu.2026.1967647

**Published:** 2026-10-26

**Authors:** Ting Peng, Xue He

**Affiliations:** Health Management Center, The Third Xiangya Hospital, Central South University, Changsha, Hunan, China

**Keywords:** cancer-associated fibroblasts, colorectal cancer, CXCR2, immune checkpoint blockade, immune exclusion, microsatellite stability, neutrophils

## Abstract

Most microsatellite-stable or mismatch-repair-proficient colorectal cancers respond poorly to immune checkpoint monotherapy. This Mini Review examines whether C-X-C motif chemokine receptor 2 (CXCR2)-associated neutrophil recruitment and local stromal interactions contribute to immune exclusion: CD8+ T cells accumulate in stroma or at the tumor margin with limited entry into epithelial nests. We propose that, in a subset of excluded lesions, recruited neutrophils acquire local functions that impair T-cell activity or access. Human observations and experimental perturbations support parts of this model; the complete pathway has not been established as a major cause of checkpoint resistance. Recruitment dependence, tissue adaptation, and measured function provide a basis for comparing studies with different neutrophil labels. Lesions selected for mechanistic investigation would need confirmed microsatellite-stable or mismatch-repair-proficient status, spatial exclusion, and a relevant intratumoral CXCR2-associated neutrophil program. Primary tumors and metastatic sites require separate evaluation. The limited activity of navarixin plus pembrolizumab argues against unselected targeting. Combining CXCR2 inhibition, selected stromal or vascular modulation, and checkpoint blockade remains a preclinical hypothesis. Before clinical translation, comparative studies must link intratumoral target engagement to improved CD8+ T-cell access, function, and tumor control. The proposed tissue biomarkers are research tools whose predictive value requires validation.

## 1 Introduction

In colorectal cancer (CRC), microsatellite instability (MSI) describes a molecular phenotype, whereas mismatch-repair (MMR) status describes the DNA repair system, commonly assessed through MMR protein expression. Microsatellite-stable (MSS) tumors are usually MMR-proficient (pMMR), and MSI-high (MSI-H) tumors are usually MMR-deficient (dMMR). Discordant cases occur, however, so the terms are not strictly interchangeable (1). We use “MSS/pMMR CRC” for these closely related clinical groups and retain each study’s reported status when discussing its results. MSI-H/dMMR CRC can respond durably to programmed cell death protein 1 (PD-1) blockade (2, 3). By contrast, a phase 2 pembrolizumab study reported no objective responses among 18 evaluable patients with pMMR CRC (4).

Mutation burden alone cannot explain the limited response in MSS/pMMR CRC. Weak priming, suppressive myeloid activity, abnormal vessels, and stromal organization can also limit immunity. Consensus molecular subtype 4 (CMS4) is characterized by transforming growth factor beta (TGF-beta) signaling, stromal invasion, and angiogenesis (5). Human pMMR and dMMR CRCs contain distinct multicellular immune neighborhoods (6). In a genetically engineered model of metastatic colon cancer, increased TGF-beta signaling in the tumor microenvironment promoted immune evasion. Pharmacological blockade of this signaling improved T-cell access and sensitized metastatic tumors to PD-1/PD-L1 blockade (7). These observations warrant examining spatial constraints, although they do not establish a common mechanism in all MSS/pMMR tumors.

This review examines whether CXCR2-associated neutrophil recruitment and local stromal interactions could contribute to immune exclusion, defined as accumulation of CD8+ T cells in the stroma or at the tumor margin with limited entry into tumor epithelial nests. We hypothesize that a subset of immune-excluded MSS/pMMR lesions depends on recruited neutrophils that acquire local suppressive or remodeling functions. Recruitment, neutrophil function, and physical access are distinct processes, and the evidence supporting the links between them varies. An immune desert with few CD8+ T cells in either compartment may instead require improved antigen presentation and priming. Throughout the review, observations in primary human tumors are distinguished from metastatic, mouse, *in vitro*, and cross-cancer evidence.

## 2 CXCR2 recruitment and neutrophil states in colorectal cancer

### 2.1 Ligands and cellular sources

Human C-X-C motif chemokine receptor 2 (CXCR2) recognizes seven C-X-C chemokines containing the glutamate–leucine–arginine (ELR) motif: CXCL1, CXCL2, CXCL3, CXCL5, CXCL6, CXCL7, and CXCL8. CXCL6 and CXCL8 can also activate CXCR1. Mice lack a direct CXCL8 orthologue, so murine Cxcl1/Cxcl2–Cxcr2 experiments cannot reproduce every feature of human CXCL8–CXCR1/2 biology (8). This receptor distinction must be considered when interpreting ligand inhibition or dual-receptor inhibition.

In SMAD4-deficient CRC, human tissue associations and cell experiments support recruitment of CXCR2-positive neutrophils by tumor-derived CXCL1/CXCL8 and a chemokine feedback loop (9). Stromal CXCL8 expression is associated with stroma-rich and adverse histology in CRC and with reduced cancer-specific survival in the pMMR subgroup. Compartment-level expression, however, does not identify fibroblasts as the producing cells or establish receptor dependence (10). METTL3–BHLHE41 and YTHDF1–p65 programs promote CXCL1-associated myeloid recruitment in experimental CRC (11, 12). S100A8 was linked to a CXCL5/CXCR2 circuit affecting colon cancer-cell proliferation and invasion, but neutrophil recruitment was not directly tested (13). The recruitment evidence is stronger for individual tumor-to-myeloid interactions than for a fibroblast-derived mechanism in primary human MSS CRC.

In experiments with apoptotic CRC cell-conditioned medium, CXCL8 mediated neutrophil chemotaxis. Neutralizing CXCL8 did not abolish the prolonged neutrophil survival caused by the medium (14). CXCR2 is also expressed in tumor cells and some stromal or endothelial populations (8). In peritoneal dissemination experiments, CXCL2–CXCR2 promoted colon cancer cell adhesion through alpha-V integrin, without demonstrating a neutrophil-mediated barrier (15). Responses to systemic CXCR2 inhibitors therefore cannot automatically be attributed to neutrophils. Cell-resolved receptor localization is needed to distinguish effects on the different populations.

### 2.2 Recruitment and local shaping of neutrophil states

CXCR2-dependent recruitment alone does not show that the recruited neutrophils suppress T cells. In experimental CRC, entry of neutrophils into colorectal tumor tissue can induce angiogenic specialization (16). Xu and colleagues used density fractionation and morphology to distinguish high-density neutrophils, mature low-density neutrophils, and immature low-density neutrophils. The mature populations were CD14-negative CD11b-positive CD66b-positive CD10-positive; the immature low-density fraction was CD14-negative CD11b-positive CD66b-negative CD10-negative. Mature low-density cells inhibited T-cell proliferation, and low-density subsets reduced CD8+ T-cell-mediated killing of CRC organoids. High-density cells could instead kill CRC cells directly (17). These states also differed in CXCR2-ligand responses and extracellular trap formation. The functional evidence included CRC liver-metastasis experiments, where neutrophils interact with a hepatic stromal and immune niche. Results from established liver metastases cannot therefore be assumed to describe neutrophil function or CD8+ T-cell access in untreated primary MSS lesions. A multimodal human CRC atlas identified heterogeneous neutrophil phenotypes and interleukin-1 (IL-1)-associated neutrophil–fibroblast niches, without causally establishing the complete MSS-specific pathway (18).

The historical N1/N2 classification originated in experimental polarization studies (19). More precise descriptions of neutrophil states use maturation, activation, angiogenic programs, trap formation, and measured immune effects (20, 21). Tumor-associated neutrophil (TAN) denotes location, and low density denotes a separation property. CD15 or CD66b positivity identifies a granulocytic phenotype, not suppressive function. Classification as polymorphonuclear myeloid-derived suppressor cells (PMN-MDSCs) requires both phenotypic characterization and functional evidence of T-cell suppression (22). Human granulocytes should be resolved using combinations of CD11b, CD14, CD15/CD66b, maturation markers, and morphology. Mouse CD11b-positive Ly6G-positive Ly6C-low cells require analogous functional validation. Bulk CD11b-positive Gr-1-positive cells or human CD33 staining alone are insufficient to identify a purified PMN-MDSC population (12, 22).

Evidence of CXCR2-associated recruitment, adaptation within a tumor or metastatic niche, and a measured effector function provides a common basis for comparing these differently named populations. This is an evidence framework, not a newly defined neutrophil subset, and no cited population is required to display every feature. In the suppression branch, the relevant function is inhibition of T-cell proliferation or tumor-cell killing. In the remodeling branch, protease expression or angiogenic activity identifies candidate populations; linking them to exclusion requires a measured effect on matrix or vessels and on CD8+ T-cell entry. Neutrophil extracellular trap (NET) formation is relevant only when its biological effect has been established under the reported inducing conditions.

The studies describe overlapping functions, but not a single shared neutrophil identity. CXCR2-associated recruitment of CD66b-positive neutrophils in SMAD4-deficient CRC and niche-induced matrix metalloproteinase 14 (MMP14)/secreted phosphoprotein 1 (SPP1) angiogenic programs address different steps of the proposed model (9, 16). Mature low-density neutrophils provide evidence of T-cell suppression and ligand-responsive NET formation in CRC liver-metastasis experiments, whereas high-density neutrophils can directly kill CRC cells (17). In the same study, immature low-density neutrophils reduced CD8+ T-cell-mediated organoid killing despite lacking CXCR2 and chemotactic responsiveness. Low density, immaturity, and TAN location are therefore neither necessary nor sufficient to identify a CXCR2-dependent mechanism. The synthesis retains each study’s neutrophil identity and tested function, including studies that did not resolve a neutrophil population.

These differences matter when interpreting immune effects. Region-specific CD16-positive neutrophils were functionally linked to natural killer cell inhibition (23). In non-metastatic CRC, CD15-high neutrophil abundance was associated with granzyme K (GZMK)-high CD8+ effector-memory T cells. Coculture supported a contact-dependent state shift, but GZMK expression itself was not associated with a dysfunctional state (24). In mice, dipeptidase 1 (DPEP1) knockout altered neutrophil retention and tumor immune features; these tumors are not equivalent to clinically defined human MSI-H disease (25).

Neutrophils and their extracellular traps can also support antitumor activity. ICAM1-high/CD95-high neutrophils were identified at early colon cancer margins (26), and interferon gamma (IFN-gamma) and chemotherapy induced tumoricidal traps in defined CRC experiments (27, 28). In human resection cohorts containing pMMR and dMMR tumors, higher CD66b-positive granulocyte density was associated with better cancer-specific survival. Citrullinated histone H3-positive trap density, however, was not independently prognostic (29). Density or a single marker alone cannot distinguish these divergent functions (30, 31).

## 3 Proposed neutrophil and stromal contributions to immune exclusion

### 3.1 Stromal support and reciprocal interactions

In a three-dimensional collagen CRC coculture, cancer-associated fibroblasts (CAFs) prolonged neutrophil survival, supporting a role in neutrophil persistence. Apoptotic tumor cells supplied additional survival-promoting activity that did not depend on CXCL8, although CXCL8 contributed to chemotaxis (14). These results show an *in vitro* survival effect; they do not demonstrate *in vivo* retention or fibroblast-derived CXCR2-ligand recruitment in primary MSS CRC.

Fibroblast studies also address immune access. Thrombospondin 2 (THBS2)-positive fibroblasts were spatially associated with exclusion in primary human CMS4/MSS CRC. Mouse perturbation experiments linked THBS2 to CXCL9/CXCL10–CXCR3-dependent T-cell recruitment (32). These findings support a stromal immune mechanism; a neutrophil or CXCR2 intermediate has not been demonstrated. ZEB1-dependent fibroblast polarization altered inflammation in CRC models (33). CAF-derived WNT2 suppressed dendritic-cell-mediated immunity in experiments that included mouse CRC, whereas the human immune-association analysis involved esophageal squamous cell carcinoma (34). In colorectal liver metastasis (CRLM), tumor-derived fibroblast growth factor 19 (FGF19) induced inflammatory CAF programs that promoted neutrophil recruitment and NET formation. Complement C5a and IL-1 beta were implicated in the NET response (35). This causal evidence comes from a hepatic stromal context and cannot be treated as direct evidence in primary MSS tumors.

In human CRC, spatial IL-1-associated neutrophil–fibroblast neighborhoods support proximity and possible communication (18). They do not identify which cell initiates the niche or whether CXCR2 is required. Hypoxia and poor perfusion may influence neutrophil persistence and function. Testing the proposed feedback between recruited neutrophils and fibrovascular remodeling requires time-resolved perturbation in a defined anatomical setting.

### 3.2 Remodeling does not necessarily create a barrier

In SMAD4-deficient CRC, recruited neutrophils expressed matrix metalloproteinases MMP2/MMP9 alongside arginase 1 (ARG1) and indoleamine 2,3-dioxygenase (IDO) (9). Enzyme expression supports remodeling potential but does not measure collagen alignment, stiffness, or T-cell passage. Experimental CRC studies identified MMP14/SPP1-associated angiogenic TAN programs; neutrophil depletion also increased poorly connected vessels and avascular regions (16). Reducing neutrophils therefore need not normalize perfusion. Studies of transplanted hypoxic tissue and pancreatic islet carcinogenesis also support neutrophil-associated angiogenesis, although neither setting is primary MSS CRC (36, 37).

NETs have promoted epithelial–mesenchymal transition (38), elastase-associated extracellular signal-regulated kinase (ERK) activation (39), and tumor mitochondrial fitness in specific CRC or metastatic models (40). Their effects depend on the inducing conditions: ligand-induced NET formation in activated low-density cells favored liver metastasis (17), whereas IFN-gamma- or chemotherapy-induced NETs were tumoricidal in other experiments (27, 28). NET detection should therefore combine extracellular DNA morphology with neutrophil proteins and functional context. Citrullinated histone staining alone does not demonstrate a protumor effect.

Proteolysis can create migration paths by degrading extracellular matrix (ECM). It can also alter adhesion cues and growth-factor availability in ways that support later stromal reorganization. The net effect on CD8+ T-cell access may vary with location, substrate, timing, and repair response. Greater protease or NET abundance does not necessarily mean greater matrix stiffness or physical exclusion. To attribute a remodeling barrier to neutrophils, an intervention must link the relevant effector to a measured change in matrix or vessels and then to CD8+ T-cell entry.

### 3.3 Distinguishing physical exclusion from biochemical suppression

Physical exclusion is the failure of CD8+ T cells to reach malignant epithelium, potentially because of restrictive collagen architecture or defective vascular entry. Biochemical suppression involves impaired T-cell signaling, proliferation, or cytotoxicity through oxidants, amino-acid depletion, or other mediators. It can occur even when T cells contact tumor cells. Both processes may occur in the same lesion, but an assay demonstrating one does not establish the other. Human immune neighborhoods and the CD15-high/GZMK-high association illustrate why density alone cannot distinguish them (6, 24).

Although the tumor–stroma boundary is organized differently in pMMR and dMMR CRC, the treated immunotherapy cohort in the spatial boundary study comprised dMMR patients (41). The TGF-beta study established causal effects in a metastatic mouse model (7), whereas THBS2 perturbation addressed a fibroblast-associated recruitment mechanism (32). None of these studies directly demonstrates the complete CXCR2 ligand–neutrophil–stroma–CD8+ T-cell exclusion sequence in primary human MSS CRC. **Figure 1** distinguishes component interactions supported experimentally in their reported settings from inferred connections in the proposed model.

**Figure 1:**
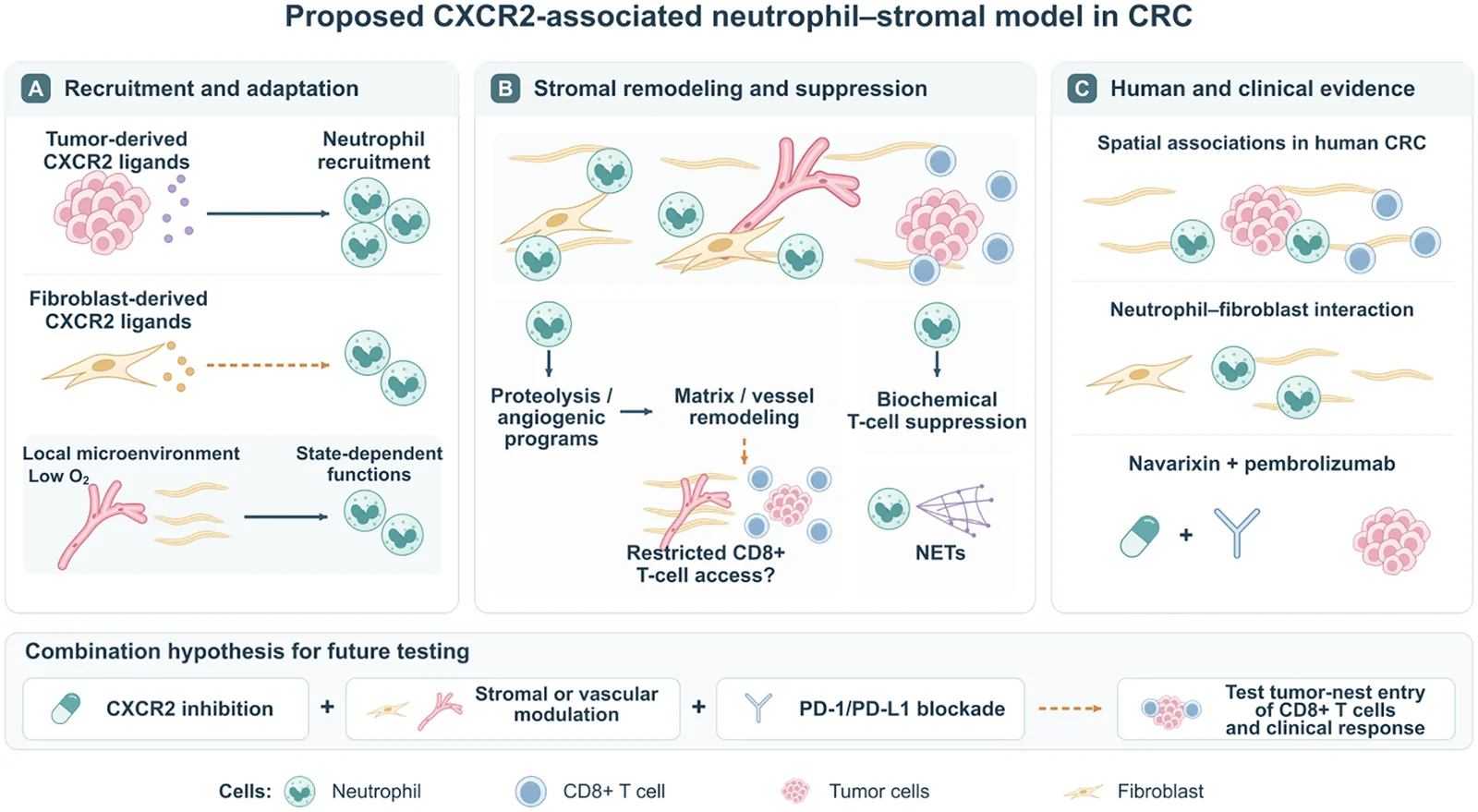
Proposed CXCR2-associated neutrophil–stromal model in CRC. **(A)** Human CRC tissue observations and cell models support recruitment by tumor-derived ligands. Experimental CRC and other models support local neutrophil adaptation. Neutrophil recruitment by fibroblast-derived CXCR2 ligands remains unproven in primary human MSS CRC. Recruitment alone does not identify suppressive neutrophils. **(B)** Proteolytic and vascular effects have experimental support. Proteolysis may loosen or reinforce barriers. Suppression has been functionally tested in defined granulocytic populations, but neither remodeling nor suppression establishes physical CD8+ T-cell exclusion. NET effects depend on stimulus and cargo. **(C)** Primary tumors and liver metastases require separate interpretation. Evidence for causal neutrophil–fibroblast interactions includes liver-metastasis models. Navarixin plus pembrolizumab showed limited activity in advanced MSS CRC, and changes in blood counts did not establish improved tumor access. Solid arrows indicate experimentally supported component interactions in their reported settings; dashed arrows indicate inferred links or proposed interventions. The complete pathway remains unproven in primary human MSS CRC, and the triple combination has not been validated in MSS CRC. Tumor-nest CD8+ T-cell entry and clinical response are outcomes to test.

The component evidence represented in **Figure 1** includes tumor-ligand recruitment supported by CRC tissue observations and cell experiments, local neutrophil adaptation in experimental CRC, and hepatic CAF-associated NET formation in CRLM models. Matrix and vascular effects do not by themselves establish physical CD8+ T-cell exclusion. The proposed therapeutic combination requires separate validation of target engagement, T-cell access, and T-cell function.

## 4 Possible contributions to checkpoint resistance

### 4.1 Functionally demonstrated immune suppression

ARG1-associated arginine depletion, reactive oxygen species (ROS), and IDO-associated tryptophan metabolism are candidate suppressive mechanisms. Assessing these mechanisms requires evidence of function beyond protein expression. In the SMAD4 study, ARG1/IDO expression in tumor-infiltrating neutrophils indicated suppressive potential; the expression data did not establish T-cell suppression (9). METTL3 and YTHDF1 studies provide mechanistic links between tumor chemokine programs and myeloid-mediated immune effects (11, 12). In the YTHDF1 study, suppression was tested in bulk CD11b-positive Gr-1-positive MDSCs rather than a purified PMN-MDSC population. Pyroptosis-associated CXCL2 signaling was linked to MDSC recruitment in CRC models (42). A microbial-metabolite-associated CXCL3 pathway recruited PMN-MDSCs and suppressed CD8+ T-cell activity (43). These results support model-specific suppression and do not make all TANs equivalent to PMN-MDSCs.

Short-range biochemical effects may contribute to resistance alongside restricted access. Identifying the dominant mechanism in an individual lesion requires more than the presence of ARG1, IDO, ROS-associated signatures, or NETs. Paired phenotypic and functional assays should test whether the relevant cells inhibit CD8+ T-cell proliferation or tumor-cell killing. These assays should also determine whether suppressive activity changes independently of spatial redistribution.

### 4.2 Spatial constraints on checkpoint activity

PD-1/programmed death ligand 1 (PD-L1) blockade can release inhibitory signaling while inadequate tumor access or priming persists. In CRC models, IL-17A blockade (44), interference with YTHDF1-associated myeloid recruitment (12), and NET inhibition improved anti-PD-1 activity under specific experimental conditions (45). CXCR2-directed trafficking interventions also enhanced checkpoint effects in rhabdomyosarcoma and pancreatic cancer models (46, 47). These findings support a preclinical rationale. They do not establish clinical CXCR2-dependent spatial resistance in MSS CRC.

Prospective studies should classify lesions as immune-desert, excluded, or inflamed before treatment. Defining exclusion requires epithelial segmentation and compartment-specific CD8+ T-cell measurements; a low whole-section count is insufficient. Anatomical site and prior therapy must remain part of the analysis, because liver metastases and primary tumors may involve different stromal mechanisms.

## 5 Therapeutic hypotheses and how to test them

### 5.1 Inhibiting recruitment

Selective CXCR2 inhibition, dual CXCR1/2 inhibition, and ligand neutralization differ in biological scope. Ligand redundancy may limit single-ligand approaches. Receptor inhibitors can affect several cellular compartments and beneficial neutrophil functions (8, 31). Preclinical trafficking studies support testing these approaches (46, 47), but a fall in circulating neutrophils does not show that a suppressive tumor population has been removed. Intratumoral receptor expression, ligand source, neutrophil abundance, and state should each be measured. Neutropenia occurred with navarixin plus pembrolizumab, although the trial reported no increased risk of neutropenic fever or infections (48).

### 5.2 Modulating neutrophil function

Interventions directed at neutrophil states might target tissue adaptation, metabolism, or NET formation (21, 31). Studies of microbiota-associated regulatory neutrophils (49) and of combined ferroptosis induction with MDSC blockade in mouse hepatocellular carcinoma and CRC liver-metastasis models (50) show that myeloid functions can be modified experimentally. Their distinct settings do not validate a common treatment for MSS CRC. Peptidylarginine deiminase 4 (PAD4) inhibition or deoxyribonuclease (DNase) treatment warrants testing only where detrimental NET activity has been established. Tumoricidal NETs and favorable granulocyte associations reinforce the need to select cells by function (27, 29). Patient-derived cocultures could distinguish effects on CD8+ T-cell function, matrix remodeling, and direct tumor killing.

### 5.3 Clinical evidence and combination hypotheses

Clinical combination studies help frame the treatment hypothesis, but have not validated CXCR2 targeting. The randomized METIMMOX study did not improve its primary progression-free survival (PFS) endpoint (51). In a small phase 1 regorafenib–ipilimumab–nivolumab study, responses were concentrated in patients without liver metastases (52). Pembrolizumab–binimetinib–bevacizumab and nivolumab–relatlimab studies also failed their prespecified primary efficacy criteria despite occasional responses (53, 54). Chemoimmunotherapy observations vary with the regimen and population (55). Whether vascular modulation restores access, or a CXCR2-containing combination improves outcomes, remains unresolved.

The phase 2 navarixin study enrolled 40 patients with previously treated advanced MSS CRC, who received pembrolizumab plus navarixin at 30 mg (n = 19) or 100 mg (n = 21) daily. MSS CRC-specific median PFS was 1.8 months (95% confidence interval [CI], 1.0–2.0) and 1.9 months (95% CI, 1.6–2.0), respectively. One partial response gave a pooled objective response rate (ORR) of 2.5% (48). Patients were randomized between navarixin doses; there was no pembrolizumab-only control. The trial stopped for futility and collected no tumor biopsies. The fall in blood neutrophils could therefore not establish intratumoral remodeling. These findings constrain efficacy claims and leave the spatial model unvalidated.

Human evidence supports lesion-specific relevance, while the pathway’s contribution to resistance remains uncertain. Stromal CXCL8 expression is associated with adverse histology and poorer survival in univariate analyses of human CRC, and SMAD4 loss is associated with peritumoral CD66b-positive neutrophil accumulation (9, 10). These patient observations support the recruitment hypothesis but do not establish a cause of checkpoint resistance. Spatial associations involving THBS2-positive fibroblasts in primary CMS4/MSS CRC provide separate evidence for stromal exclusion (32). Together, these findings warrant testing a neutrophil contribution in excluded lesions. Whether the chemokine, neutrophil, and fibroblast observations form one causal sequence remains to be determined.

Determining whether the pathway is a major contributor requires prospective estimates of its frequency in immune-excluded MSS/pMMR CRC. Studies would also need to determine whether disrupting it reverses resistance relative to competing causes, including inadequate priming and neutrophil-independent stromal exclusion. Neither frequency estimates nor validated treatment-selection rules are available. The navarixin findings show why MSS status alone and reduced circulating neutrophil counts are insufficient grounds for selecting a CXCR2-containing regimen. The failed doublet provides no evidence that adding a third agent would restore efficacy.

The proposed triple treatment would use CXCR2 inhibition to reduce detrimental neutrophil recruitment, selected stromal or vascular modulation to improve CD8+ T-cell access, and checkpoint blockade to support tumor-reactive T-cell activity. This remains a preclinical hypothesis rather than a recommended regimen or a trial-ready strategy. The second intervention must address the mechanism demonstrated in the lesion. Fibroblast-associated TGF-beta or THBS2 signaling and impaired vascular entry are not interchangeable targets (7, 32). Antiangiogenic treatment cannot be assumed to normalize perfusion, particularly when neutrophil-directed intervention can increase avascularity (16).

Before clinical translation, site-matched, immunocompetent CRC models should compare untreated controls, each single agent, all doublets, and the triple combination under a prespecified dosing schedule. Patient-derived cocultures could test whether CXCR2-dependent recruitment and detrimental neutrophil function occur in the same biological setting. Cell-resolved perturbations, with rescue experiments where feasible, should distinguish effects of neutrophil CXCR2 from effects on tumor cells or other stromal populations. The triple combination would need to improve both tumor control and the proposed intermediate steps beyond the relevant doublets. Increased intranest CD8+ T-cell density without improved killing, or tumor regression without reversal of the proposed mechanism, would require a different interpretation. These studies should precede a biomarker-enriched early clinical trial.

### 5.4 Candidate populations and research biomarkers

The most relevant patients would have confirmed MSS/pMMR CRC and an immune-excluded lesion containing an intratumoral CXCR2-associated neutrophil program and a demonstrable stromal or vascular constraint. Stroma-rich or CMS4 tumors warrant investigation, but CMS4 alone cannot establish neutrophil dependence or treatment eligibility. SMAD4 loss or elevated CXCL8 may support the recruitment hypothesis but cannot replace lesion-level assessment. Immune-desert lesions fall outside the immediate access-restoration hypothesis. MSI-H/dMMR lesions require separate analysis because their checkpoint sensitivity and spatial immune organization differ.

Untreated primary tumors should form a separate anatomical and treatment stratum. Previously treated metastases, including liver and peritoneal lesions, need separate strata documenting prior therapy and the sampled site. Liver-metastasis evidence may support a hepatic mechanism, but cannot define eligibility for primary-lesion studies. Responses concentrated in patients without liver metastases in a small regorafenib-based trial do not establish liver-metastasis status as a predictor for a CXCR2-containing combination (52). No clinical population has yet been shown to benefit from the proposed triple treatment.

Candidate baseline enrichment biomarkers should assess co-occurrence of the proposed steps in the same lesion. Epithelial segmentation and CD8+ T-cell mapping would identify stromal or marginal accumulation with limited epithelial-nest entry. Cell-resolved ligand measurements and granulocyte CXCR2 localization would assess recruitment plausibility. Neutrophil morphology and maturation markers would characterize the population; ex vivo suppression or context-specific remodeling assays would test its detrimental function. Fibroblast programs, collagen architecture, or impaired perfusion would guide the choice of the second intervention. These proposed research criteria need reproducible assays and prospectively established thresholds. Bulk CXCL8 expression, CMS4 status, peripheral neutrophil counts, and the neutrophil-to-lymphocyte ratio alone cannot demonstrate the linked mechanism or predict treatment benefit.

If comparative preclinical evidence supports an early trial, paired biopsies should sample the same lesion before and during treatment. Primary tumors and metastatic sites should be analyzed separately. Intratumoral pharmacodynamic endpoints should assess CXCR2 target engagement or reduced CXCR2-dependent recruitment, changes in the identified neutrophils’ function, and effects of the selected stromal or vascular intervention. Candidate access endpoints include tumor-nest CD8+ T-cell density, the proportion of CD8+ T cells within epithelial nests, their distance to the nearest tumor cell, and the ratio of intranest to stromal CD8+ T-cell density. Collagen organization, fibroblast states, vessel structure, hypoxia, and perfusion should be assessed as appropriate for the intervention. Separate functional assays should measure T-cell proliferation and tumor-cell killing. These on-treatment measures test the mechanism and are not validated baseline predictors. Clinical evaluation must link target engagement, inward T-cell redistribution, and improved function to radiographic response and PFS. Blood counts and serum chemokines are supportive measures. Predictive utility requires prospective validation and an appropriate treatment comparator. **Table 1** summarizes the selected evidence and its population-specific limitations.

**Table 1:** Selected evidence for the proposed CXCR2-associated neutrophil–stromal model.

Study	Anatomical site	MSI/MMR status	Model and population	Neutrophil identity and tested function	Evidence and finding	Main limitation
Ogawa (9)	Primary CRC and peritumoral stroma	Human result not MSI/MMR-stratified	Human tissue and cell coculture; chemotaxis assays	CD66b-positive peritumoral TANs; CXCR2-associated chemotaxis. ARG1/IDO and MMP2/MMP9 expression indicates functional potential, but T-cell suppression was not demonstrated.	Human association; *in vitro* CXCL1/CXCL8–CXCR2 recruitment and feedback	No demonstrated stromal–CD8+ T-cell exclusion sequence in primary MSS CRC
Pennel (10)	Primary CRC; matched liver metastases	pMMR/dMMR assessed; survival association in pMMR	Human spatial RNA and histology	CD66b-positive granulocyte infiltrates; CXCR2-positive immune cells identified mainly as neutrophils by histology; systemic neutrophil counts. No isolated-cell suppression assay.	Association of stromal CXCL8 with stroma-rich, adverse histology	Fibroblast source and CXCR2-dependent exclusion unproven
Schimek (14)	CRC cell models; CG08 from peritoneal-metastatic ascites	Not MSI/MMR-stratified	Human collagen coculture; migration and survival assays	Healthy-donor blood and CRC ascites neutrophils. Apoptotic CRC-conditioned medium induces CD66b-high/CD11b-high/CD62L-low activation. Migration, survival and macrophage effects tested; no CD8+ T-cell exclusion assay.	*In vitro*: CXCL8-mediated chemotaxis; neutralization did not abolish prolonged survival	CXCL8-dependent survival or retention unproven in primary human CRC
Bui (16)	Colonic tumors; xenografts	Human analysis not MSI/MMR-stratified	AOM/DSS mice; CRC xenografts; vascular assays	Mouse Ly6G-high/CD11b-high/Lyz2-high TANs with MMP14/SPP1 angiogenic programs. Endothelial and vascular effects tested; suppressor identity not established.	Experimental remodeling; intervention could increase collagen and avascularity	Remodeling does not establish improved perfusion or CD8+ T-cell entry
Xu (17)	CRC blood/tissue; liver-metastasis models	No MSS-specific human causal result	Human cells/organoids and mice; proliferation/killing assays	Density/morphology-defined HDNs, mature LDNs and immature LDNs. Mature LDNs suppress T-cell proliferation; both LDN types reduce CD8+ T-cell-mediated killing; HDNs can kill CRC cells. Immature LDNs lack CXCR2 responsiveness.	Experimental: state-dependent NET formation, T-cell suppression and tumor killing	Low density alone does not define suppression; primary MSS barrier unproven
Iwane (32)	Primary human tumor front; mouse tumors	Human CMS4/MSS versus CMS1/MSI	Spatial tissue analysis; fibroblast and chemokine perturbation	No neutrophil subset tested; THBS2-positive fibroblasts and CD8+ T-cell recruitment were the mechanistic focus.	Human spatial association; mouse stromal immune effects involving CXCL9/10–CXCR3	No demonstrated neutrophil/CXCR2 intermediate
Li (35)	Paired primary CRC/liver metastases; hepatic models	Human mechanism not MSI/MMR-stratified	Human tissue; stellate-cell coculture; mouse liver colonization	Healthy-donor blood neutrophils isolated by Ficoll; NET structures assessed *in vitro* and in hepatic models. No subset defined by maturation or density; CD8+ T-cell exclusion untested.	Hepatic experiments: CAF signaling supported NET formation	CXCR2 dependence and physical CD8+ T-cell exclusion untested
Armstrong (48)	Previously treated advanced CRC	MSS cohort, n = 40	Phase 2; randomized navarixin doses plus pembrolizumab	Peripheral absolute neutrophil counts; no intratumoral neutrophil identity or functional assessment.	Clinical: ORR 2.5%; median PFS 1.8 months (30 mg) and 1.9 months (100 mg)	No pembrolizumab-only control or tumor biopsies; barrier reversal untested

Study-specific neutrophil identity and tested function are distinguished from evidence for the proposed pathway. MSI/MMR status refers to the cited analysis. The complete pathway remains unproven in primary human MSS CRC.

AOM/DSS, azoxymethane/dextran sodium sulfate; HDN/LDN, high-/low-density neutrophil; Ly6G, lymphocyte antigen 6 complex locus G; RNA, ribonucleic acid. CG08 is a human CRC cell line.

## 6 Discussion and future perspectives

CXCR2-associated recruitment of locally adapted neutrophils may contribute to resistance in a biologically defined subset of immune-excluded MSS/pMMR CRC lesions. Human observations support the clinical relevance of this hypothesis, and experimental perturbations support selected component mechanisms. How often the complete pathway occurs, how much it contributes relative to competing mechanisms, and whether a triple intervention improves clinical outcomes remain unresolved. Comparing neutrophil populations by recruitment dependence, local adaptation, and tested effects on T-cell function or access preserves the distinctions between differently named subsets. Selecting a detrimental population also requires attention to beneficial neutrophil and NET effects.

The hypothesis should be tested in specimens that allow the proposed biological steps to be evaluated together. Primary CRC specimens need documented MSI/MMR assay results, treatment history, and epithelial boundaries; liver-metastasis specimens address a hepatic mechanism. Studies should identify ligand-producing cells, localize CXCR2 to the relevant granulocyte population, characterize its morphology and phenotype, and measure its proposed effector function. Spatial imaging should determine whether CD8+ T cells remain outside epithelial nests. Perturbation experiments should then test whether neutrophils cause biochemical suppression, altered matrix or vascular access, or both. Human atlases can guide the choice of cells and neighborhoods to investigate (18), while tumor-front studies provide complementary spatial endpoints (32, 41). The next step is comparative preclinical validation of the linked mechanism and each treatment component. The present evidence does not justify an unselected clinical triple-combination trial.
